# Direct Evidence of Brown Adipocytes in Different Fat Depots in Children

**DOI:** 10.1371/journal.pone.0117841

**Published:** 2015-02-23

**Authors:** Denise Rockstroh, Kathrin Landgraf, Isabel Viola Wagner, Julia Gesing, Roy Tauscher, Nicole Lakowa, Wieland Kiess, Ulf Bühligen, Magdalena Wojan, Holger Till, Matthias Blüher, Antje Körner

**Affiliations:** 1 Center for Pediatric Research Leipzig, University Hospital for Children & Adolescents, Department of Women’s and Child Health, University of Leipzig, Leipzig, Germany; 2 Integrated Research and Treatment Center (IFB), University of Leipzig, Leipzig, Germany; 3 Department of Medicine, Division of Endocrinology, University of Leipzig, Leipzig, Germany; 4 Department of Pediatric Surgery, University of Leipzig, Leipzig, Germany; 5 Department of Orthopaedic Surgery, University of Leipzig, Leipzig, Germany; 6 Department of Pediatric and Adolescent Surgery, Medical University Graz, Graz, Austria; University of Bonn, GERMANY

## Abstract

Recent studies suggested the persistence of brown adipocytes in adult humans, as opposed to being exclusively present in infancy. In this study, we investigated the presence of brown-like adipocytes in adipose tissue (AT) samples of children and adolescents aged 0 to 18 years and evaluated the association with age, location, and obesity. For this, we analysed AT samples from 131 children and 23 adults by histological, immunohistochemical and expression analyses. We detected brown-like and UCP1 positive adipocytes in 10.3% of 87 lean children (aged 0.3 to 10.7 years) and in one overweight infant, whereas we did not find brown adipocytes in obese children or adults. In our samples, the brown-like adipocytes were interspersed within white AT of perirenal, visceral and also subcutaneous depots. Samples with brown-like adipocytes showed an increased expression of *UCP1* (>200fold), *PRDM16* (2.8fold), *PGC1α* and *CIDEA* while other brown/beige selective markers, such as *PAT2*, *P2RX5*, *ZIC1*, *LHX8*, *TMEM26*, *HOXC9* and *TBX1* were not significantly different between UCP1 positive and negative samples. We identified a positive correlation between *UCP1* and *PRDM16* within UCP1 positive samples, but not with any other brown/beige marker. In addition, we observed significantly increased *PRDM16* and *PAT2* expression in subcutaneous and visceral AT samples with high *UCP1* expression in adults. Our data indicate that brown-like adipocytes are present well beyond infancy in subcutaneous depots of non-obese children. The presence was not restricted to typical perirenal locations, but they were also interspersed within WAT of visceral and subcutaneous depots.

## Introduction

Adipose tissue (AT) exists in two forms, white (WAT) and brown adipose tissue (BAT), which differ in terms of morphology, function, abundance, origin, and regulation. In contrast to WAT, BAT stores little fat but is crucial for nonshivering thermogenesis through the action of uncoupling protein 1 (UCP1) [1]. BAT is biologically characterized by multilocular lipid droplets, abundance of mitochondria and the expression of the BAT-specific *UCP1* [2]. Considering that obesity results from a mismatch in energy supply and utilization, BAT has recently gained scientific interest for its functional capacity to burn excess energy [3,4].

Earlier postmortem studies in humans indicated that BAT is established at the fifth week of gestation, peaks at the time of birth, but declines shortly after birth [5–7]. Recent studies, mainly employing positron emission tomography (PET) scanning techniques using [^18^F]fluorodeoxyglucose uptake, provided evidence that BAT is present in both pediatric and adult subjects [3,8–12]. So far, it is controversial whether BAT activity is related to body weight in children, with some studies describing an inverse relationship between BMI and BAT activity, while others found no significant differences in BMI between children with and without functional BAT [13–15]. One group reported that the volume of BAT increases during puberty, which is assumedly related to the gain in muscle mass [15,16].

Lineage tracing studies have specified two types of brown adipocytes. The so called “classical brown” adipocytes originate from a common lineage of myogenic factor 5 expressing *(MYF5)* precursor cells and are frequently found in the interscapular region in both rodents and humans [17–20]. Recently, a new type of brown-like adipocytes has been described, termed “beige adipocytes” [21]. These cells, that derive from *MYF5* negative precursor cells, can be found interspersed within various WAT depots and exhibit a different gene expression pattern as compared to classical brown adipocytes [21,22]. In response to different stimuli they are supposed to transform from cells having a white phenotype into cells having a brown-like phenotype, including multilocular lipid droplets and *UCP1* expression [23]. A number of marker genes have been suggested to distinguish white, beige, and brown adipocytes. These include *leptin* (*LEP)*, *Asc-type amino acid transporter 1 (ASC1)* for white adipocytes, *adiponectin* (*ADIPOQ*) as a general AT marker; *Transmembrane Protein 26 (TMEM26)*, *Homeobox C9 (HOXC9)* and *T-box 1 (TBX1)* for beige adipocytes; *UCP1*, *PR domain containing 16 (PRDM16)*, *Proton assistant amino acid transporter-2 (PAT2)*, *Purinergic receptor P2X*, *ligand-gated ion channel 5 (P2RX5)* for markers of both brown and beige adipocytes; and *Zinc finger protein of the cerebellum 1 (ZIC1)* and *LIM homeobox 8 (LHX8)* for classical brown adipocytes [20,24,25].

So far only few studies have systematically investigated the presence and classification of brown adipocytes at the level of the AT on histological and molecular level in humans, particularly in children [14]. Given the potential importance of BAT in energy homeostasis and the development of obesity and considering that development of obesity starts at childhood age, the investigation of BAT in children is of interest [26].

In this study, we aimed to evaluate the presence of brown adipocytes in AT samples of children across a wide range of age and characterize the association with the development of obesity, age and anatomical location at the molecular and histological level.

## Materials and Methods

### Subjects and samples

We have established the Leipzig Adipose Tissue Childhood cohort, encompassing AT samples from 163 white children, and additionally from 52 adults [27]. Obtained tissue samples weighed 0.04g to 16.4g and were collected during elective surgery. After applying exclusion criteria which likely affect body composition and/or metabolism like *1)* type 1 or type 2 diabetes, *2)* generalized inflammation, *3)* cardiovascular diseases, *4)* malignant diseases and *5)* genetic syndromes and 6*)* chronic immobility or cerebral palsy, 131 children (87 lean, 16 overweight, 28 obese) and 23 healthy adults (9 lean, 9 overweight, 5 obese) were included into analyses. In subsequent analyses, overweight and obese patients have been grouped together and are referred to as obese. Anthropometric and demographic data, including age, gender, height, weight, waist, medical history and the mean outside temperature during the last three days before surgery were recorded. BMI was standardized to age and gender-specific centiles applying German reference data and are given as BMI standard deviation score (SDS) [28]. A cut off of 1.28 and 1.88 SDS (corresponding to the 90th and 97th centile) defines overweight and obesity in children. Subject characteristics are provided in Table 1. AT biopsies were obtained from children undergoing orthopedic surgery (knee or hip, n = 68), herniotomy or orchidopexy (inguinal area, n = 43), nephrectomy (perirenal area n = 2), pyeloplasty (perirenal area, n = 4), kidney cysts resection (subcutaneous area, n = 2), abdominal surgery (subcutaneous area, n = 6), thoracic surgery (visceral area, n = 1; subcutaneous area, n = 3), back surgery (subcutaneous area, n = 1), and splenectomy (visceral area, n = 1). Biopsies from adults were collected during orthopedic surgeries (knee or hip area, n = 23). The study was approved by the local ethics committee (Reg.No: 265-08, 265-08-ff) and is registered in the National Clinical Trials database (NCT02208141).

**Table 1:** Characteristics of the Leipzig Adipose Tissue Childhood Cohort.

	CHILDREN			ADULTS		
	Lean	Obese	*p*	Lean	Obese	*p*
N	87	44		9	14	
female/male [n]	34/53	19/25	0.651	7/2	6/8	0.099
Age [years]	6.9 ±0.6	10.8 ±0.7	**<0.001**	55.4 ±6.7	61.2 ±3.8	**0.369**
	(0.1–18.2)	(0.4–18.3)		(27.5–78.1)	(37.8–89.2)	
BMI-SDS	-0.4 ±0.1	2.2 ±0.1	**<0.001**	-0.5 ±0.2	0.4 ±0.2	**<0.016**
	(-2.5–1.2)	(1.3–3.6)		(-1.6–0.2)	(-0.5–1.9)	
BMI [kg/m²]	16.6 ±0.2	26.6 ±0.7	**<0.001**	23.9 ±0.4	28.4 ±0.8	**<0.001**
	(12.5–23)	(17.8–34.5)		(21.8–25)	(25.5–35.1)	
PH	<2	>2	**0.001**			
	(1–5)	(1–6)				
Adipocyte area [μm²]	3605.0 ±223.6	5206.9 ±316.0	**<0.001**	6901.6 ±271.2	5873.3 ±319.3	0.072
	(407–10200)	(680–8557)		(6162–7571)	(3626–7558)	
**Depots**						
Subcutaneous	80	43		9	14	
Perirenal	5	1				
Visceral	2					

Data are given as mean ± SEM (range). For gender, statistical significance was analysed by chi square test. Statistical significance for differences between lean and obese individuals was determined by Students t-test. Significant *p*-values are indicated in bold. BMI, body-mass index; SDS, standard deviation score; PH, pubertal stage.

In accordance to our childhood samples, we analyzed a further distinct adult cohort. In this cross-sectional study, paired omental and subcutaneous AT samples were obtained from extensively characterized patients, who underwent an elective bariatric surgery or surgery for diverticulitis, and cholecystolithiasis [29]. To analyse these data in a similar way as the childhood cohort, we compared the expression of brown and/or beige markers between the 10% with highest (UCP1 High) and the lowest 10% (UCP1 Low) of *UCP1* mRNA. Subject characteristics for this subcohort are provided in Table 2. For each participant, demographic and anthropometric data, concomitant medication before surgery and routine laboratory results were recorded. The study was approved by the local ethics committee (Reg.No. 031-2006 and 017-12-23012012). All participants and/or their parents gave written informed consent.

**Table 2:** Characteristics of adult subjects with low or high *UCP1* expression levels in AT.

	SUBCUTANEOUS SAMPLES			VISCERAL SAMPLES		
	Low UCP1	High UCP1	p-value	Low UCP1	High UCP1	p-value
N	48	48		48	48	
female/male [n]	35/13	29/19	0.194	38/10	28/20	**0.027**
Age [years]	47.3±1.6	53.1±2.2	**0.033**	49.7±1.7	51.1±1.6	0.561
	(26.2–73.0)	(24.5–86.8)		(26.4–80.5)	(24.5–77.2)	
BMI [kg/m²]	48.1±1.5	43.7±2.1	0.091	45.2±1.3	43.3±1.7	0.370
	(18.8–73.6)	(19.0–88.8)		(18.8–59.3)	(19.2–67.4)	
*UCP1*expression	0.6±0.05	278.5±68.3	**<0.001**	0.6±0.05	372.0±90.2	**<0.001**
	(0.0–1.2)	(42.4–2970.7)		(0.0–1.105)	(58.3–2864.9)	

Data are given as mean ± SEM (range). For gender, statistical significance was analysed by chi square test. Statistical significance for differences between groups was determined by Students t-test. Significant *p*-values are indicated in bold. BMI, body-mass index.

### Immunohistochemical and morphometric analyses of adipose tissue samples

For morphological analyses AT was fixed in 4% paraformaldehyde overnight at 4°C, dehydrated, paraffin embedded and sectioned (12μm). Adipocyte area was digitally determined in hematoxylin-eosin stained sections taken from 3 different locations (x100 magnification) and mean cell area was calculated from 100 cells per sample using Image J software (public domain, National Institutes of Health, Maryland, USA) [30,31]. For immunohistochemistry, antigen unmasking was performed in citrate buffer pH 9.0 (Target Retrieval Solution, DAKO, Glostrup, Denmark) and endogenous peroxidase was blocked by incubation with 3% hydrogen peroxide in Tris buffer (pH 7.56) for 10min. Sections were incubated with primary polyclonal rabbit UCP1 antibody (1:500; ab10983, Abcam, Cambridge, England) antibody for 30min. As a negative control primary antibody was omitted. Visualization was achieved by HRP labelled goat anti-rabbit immunoglobulin (1:200, P0448, DAKO) and Envision dual link system-HRP (DAKO).

### Gene expression analyses

Total RNA was extracted from liquid nitrogen frozen AT samples using RNeasy Lipid Tissue Mini Kit (Qiagen, Hilden, Germany). RNA was reverse transcribed using the M-MLV Reverse Transcriptase Kit (Life Technologies, Darmstadt, Germany) and random hexamer [p(dN)_6_] primers (Roche, Basel, Switzerland). *UCP1*, *ZIC1*, *PRDM16*, *PAT2*, *P2RX5*, *ZIC1*, *LHX8*, *TMEM26*, *TBX1*, *HOXC9 LEP*, *ACS1* and *ADIPOQ* expression were quantified by quantitative *real-time* PCR with the ABI 7500 Sequence Detection System (Applied Biosystems, Darmstadt, Germany). Target gene expression of samples from children was normalized to the mean of the three housekeeping genes: *β-actin* (*ACTB)*, *TATA-box-binding protein* (*TBP*) and *Hypoxanthine phosphoribosyltransferase 1 (HPRT1)*. Target gene expression of the additional adult cohort was normalized to the housekeeping gene *ribosomal protein L27* (*RPL27*). Primer and probes are given in S1 and S2 Tables.

For evaluation of a broader spectrum of genes of interest, the Illumina BeadChip Microarray was employed in 112 whole AT samples. Before microarray analysis, RNA integrity and concentration was examined on an Agilent 2100 Bioanalyzer (Agilent Technologies, Palo Alto, CA, USA) using the RNA 6.000 LabChip Kit (Agilent Technologies) according to the manufacturer’s instructions. 250ng RNA per sample were precipitated in ethanol with GlycoBlue (Invitrogen) as carrier and dissolved at a concentration of 100–150ng/μl prior to probe synthesis using the TargetAmp- Nano Labeling Kit for Illumina Expression BeadChip (Epicentre Biotechnologies, Madison, WI, USA). 750ng of cRNA were hybridized to Illumina HT-12 v4 Expression BeadChips (Illumina, San Diego, CA, USA) and scanned on the Illumina iScan instrument according to the manufacturer’s specifications. Raw data of all 47,323 probes were extracted by Illumina GenomeStudio. Expression values were quantile normalized and background subtracted.

### Statistical analyses

Gaussian distribution of parameters was assessed by Kolmogorov-Smirnov test and QQ plots. Non-Gaussian distributed data were log-transformed (log) before analyses. Categorical variables were analyzed by χ^2^ test. Significant differences between groups were analysed by two-sided Student’s *t* test or one-way ANOVA with post-hoc Dunnett’s test. Quantitative associations were examined with parametric Pearson correlation test. Analyses were performed using the Statistica 7.1 software package (Stat-Soft, Tulsa, OK).

## Results

### Identification of brown adipocytes in adipose tissue samples of children

Histologically, we identified islets of multilocular fat cells, suggestive for brown-like adipocytes, interspersed within typical unilocular WAT in eight out of 131 samples. These samples were derived from subcutaneous inguinal locations (n = 3) coming from lean children undergoing orchidopexy or hernia repair. Perirenal samples (n = 3), coming from lean and one overweight infant having pyeloplasty, and visceral samples (n = 2), were collected during a splenectomy or a thorax surgery.

**Fig 1:**
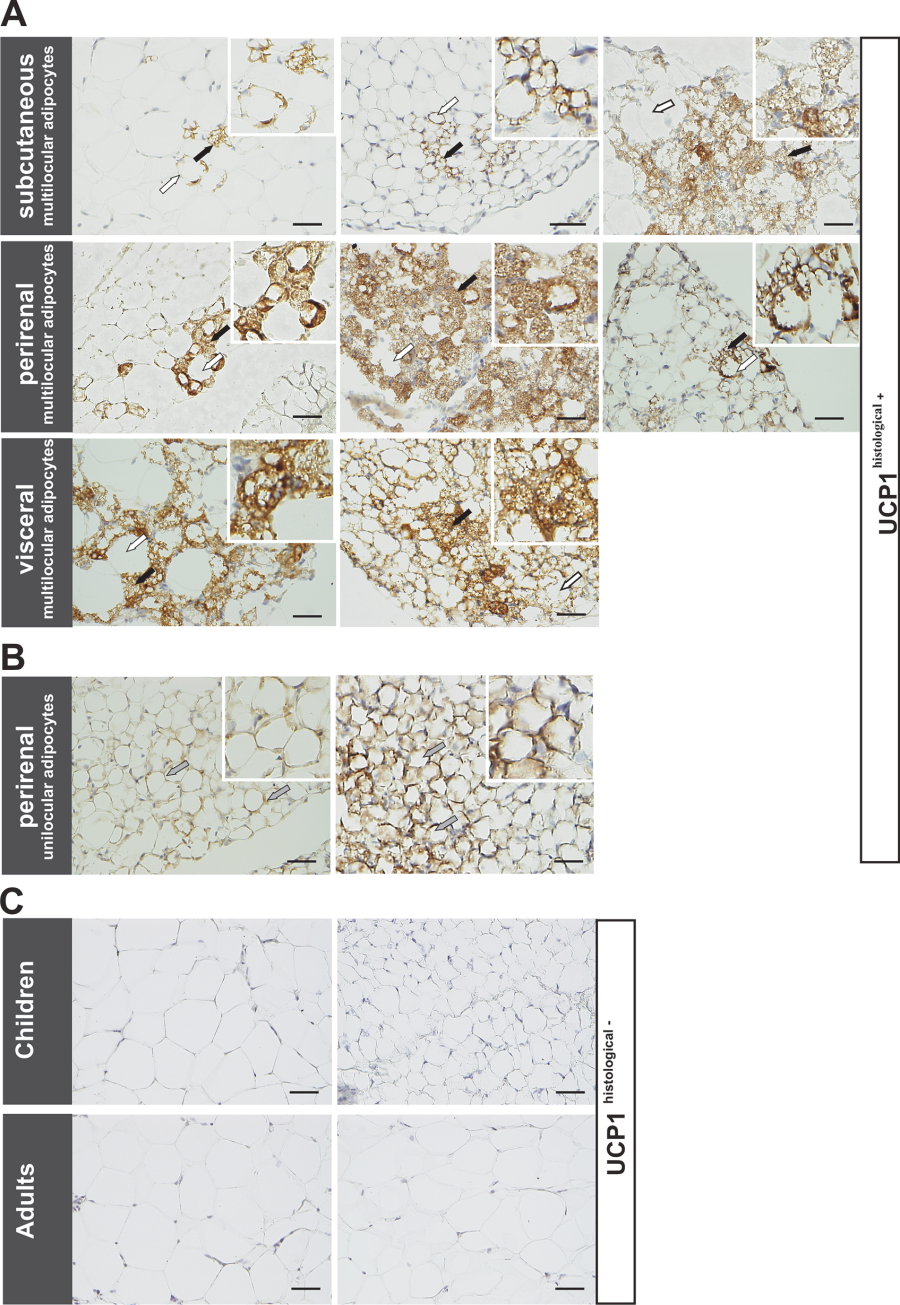
Immunohistochemical evidence of brown-like adipocytes in adipose tissue samples. A: Immunohistochemical staining of AT samples showed multilocular adipocytes, which stained positive for UCP1 (black arrows), surrounded by UCP1-negative white unilocular fat cells (white arrows) in subcutaneous (top), perirenal (middle) and visceral (bottom) AT samples of children. B: Two perirenal unilocular samples also exhibited positive UCP1 staining (grey arrows). C: Representative images of UCP1-negative subcutaneous (top, left) and perirenal (top, right) AT samples of lean children and subcutaneous samples of lean adults (bottom). Nuclei were counterstained with Mayer’s hematoxylin. Scale bars represent 50 μm in each panel.

To confirm these islets as being brown adipocytes, we performed immunohistochemical staining for the brown adipocyte specific marker UCP1. All samples with histologically multilocular adipocytes stained positive for UCP1 (Fig. 1A). In addition, two perirenal sections stained positive for UCP1, but lacked typical multilocular adipocyte morphology (Fig. 1B). These samples derived from lean children having a nephrectomy or pyeloplasty. The remaining 120 subcutaneous samples with unilocular adipocytes and one perirenal sample did not show UCP1 staining (representative pictures shown in Fig. 1C). In the following context, samples with or without positive UCP1 staining are referred to as "UCP1^histological+^" and "UCP1^histological-^".

**Fig 2:**
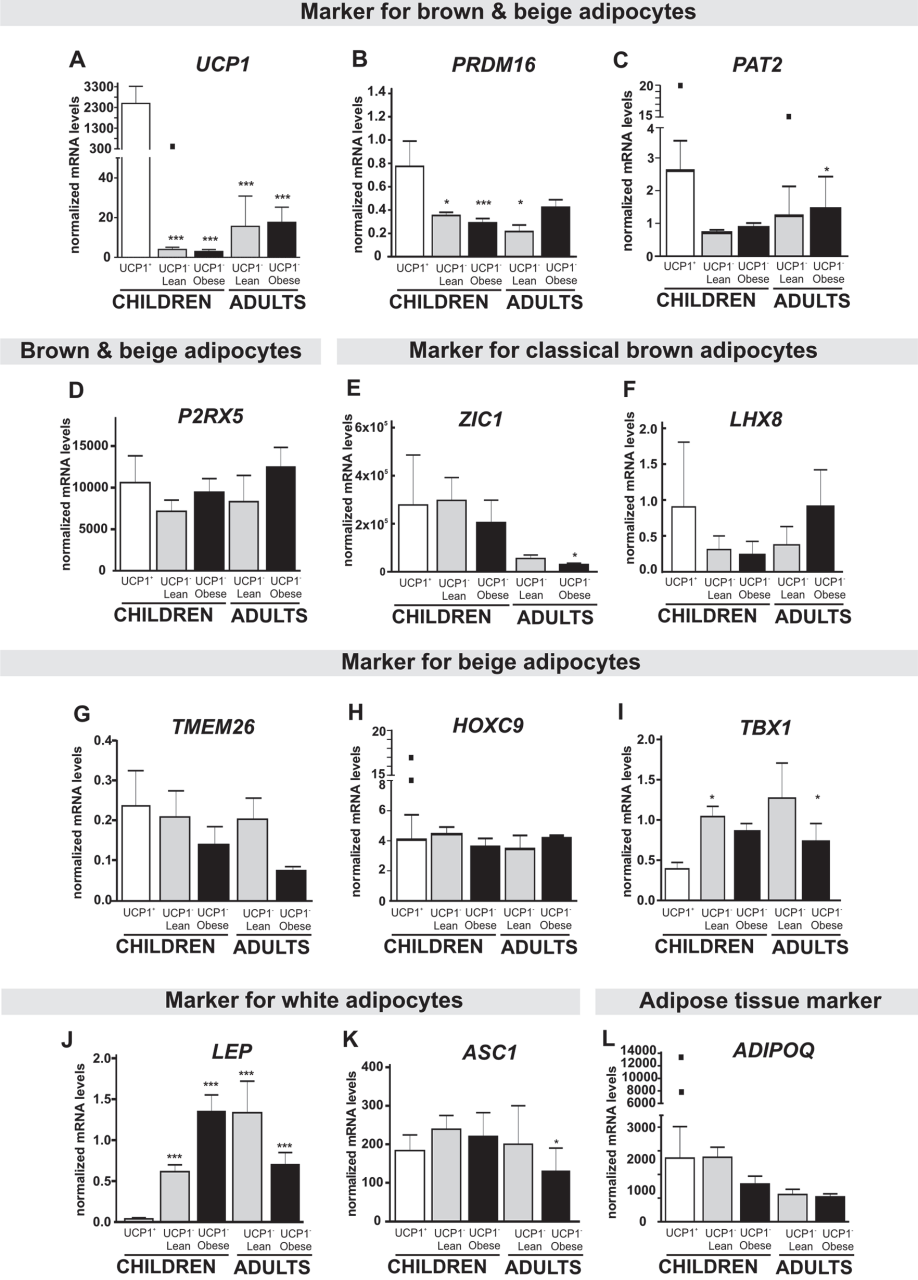
Expression of brown, beige and white adipocyte markers in adipose tissue samples of children and adults. A: *UCP1* mRNA expression was significantly increased in UCP1^histological+^ samples compared to lean and obese children as well as adult UCP1^histological-^ samples with the exception of a single outlier from an UCP1^histological-^ subcutaneous sample (indicated as black box). B: *PRDM16* was also increased in UCP1^histological+^ compared to all other UCP1^histological-^ samples. C, D, E and F: mRNA expression of *PAT2*, *P2RX5*, *ZIC1* and *LHX8* were not different in UCP1^histological+^ samples, although, one UCP1^histological+^ and one UCP1^histological-^ sample presented exceedingly high *PAT2* expression values, marked with black squares. G,H and I: mRNA expression of the beige adipocyte markers *TMEM26* and *HOXC9* was not different in UCP1^histological+^ samples, although, two UCP1^histological+^ samples (from different donors) presented exceedingly high *HOXC9* values, marked with black squares. These samples showed only islets of UCP1 positive adipocytes. *TBX1* mRNA expression was slightly increased in UCP1^histological-^ samples of lean children and obese adults compared to UCP1^histological+^ samples. J and K: The WAT marker *LEP* was significantly decreased in UCP1^histological+^ samples compared to UCP1^histological-^ samples in contrast to *ASC1*, another white specific marker. L: *ADIPOQ*, a general marker for AT was not different between UCP1^histological+^ and UCP1^histological-^ samples. Target gene expression was normalized to the mean of the three housekeeping genes: *ACTB*, *TBP* and *HPRT1*. Statistical significance was assessed by one-way ANOVA and post-hoc Dunnett’s test and was compared to UCP1^histological+^ samples and marked with *P<0.05; **P< 0.01; ***P<0.001.

On mRNA level, UCP1^histological+^ samples showed a ≥200fold increased *UCP1* expression compared to UCP1^histological-^ samples, verifying these islets as brown-like adipocytes (Fig. 2A). Of note, one subcutaneous UCP1^histological-^ sample from a hernia repair showed a strong *UCP1* expression, which was comparable to the expression in UCP1^histological+^ samples (Fig. 2A, black square). UCP1^histological+^ samples primarily originated from lean children, although we detected brown-like adipocytes in a perirenal sample of a 4 month old overweight girl (BMI-SDS 1.8). In none of the 43 subcutaneous samples from obese children we detected evidence for brown-like adipocytes. Concerning the age distribution, 60% of children with brown-like adipocytes were older than one year (seven children aged 0–2 years, two children aged 3–5 years, one child aged 10 years) (Table 3).

**Table 3:** Comparison of UCP1^histological+^ and UCP1 ^histological-^samples.

	UCP1^histological+^	UCP1 ^histological -^	*p*
N	10	121	
female/male [n]	3/7	50/71	0.48
Age [years]	2.4±1.0	8.7±0.5	**< 0.001**
	(0.34–10.7)	(0.1–18.3)	
BMI-SDS	-0.7±0.4	0.59±0.1	**0.005**
	(-2.1–1.8)	(-2.5–3.6)	
***Subcutaneous n [% of the cohort]***			
	3 [2.4%]	120 [97.6%]	
Age [years]	1.3±0.5	8.7±0.5	**0.020**
	(0.3–2.2)	(0.1–18.3)	
BMI-SDS	-1.5±0.2	0.6±0.1	**0.012**
	(-1.9– -1.1)	(-2.5–3.6)	
***Perirenal n [% of the cohort]***			
	5 [83.4%]	1 [16.6%]	
Age [years]	1.7±0.8	0.3	
	(0.4–4.7)		
BMI-SDS	-0.5±0.7	0.5	
	(-2.1–1.8)		
***Visceral n [% of the cohort]***			
	2 [100%]		
Age [years]	5.7±4.9		
	(0.7–10.7)		
BMI-SDS	-0.05±0.3		
	(-0.3–0.2)		

Data are given as mean ± SEM (range). For gender, statistical significance was analysed by Χ^2^ square test. Statistical significance for differences between groups was determined by Students t-test. Significant *p*-values are indicated in bold. BMI, body-mass index; SDS, standard deviation score.

In addition, we had access to subcutaneous AT samples of 23 healthy adults aged 27–89 years. Contrary to the observations in children, we did not observe multilocular fat cells or UCP1 specific staining indicative for brown-like adipocytes in adults (representative pictures shown in Fig. 1C).

### Molecular signature of UCP1^histological+^ adipose tissue samples

For a more detailed characterization of UCP1^histological+^ and UCP1^histological-^ samples, we evaluated mRNA expression of markers for brown, beige, or white adipocytes. *PRDM16* was more than doubled in UCP1^histological+^ samples compared to UCP1^histological-^ samples (Fig. 2B). We did, however, not observe statistically significant differences in the expression of *PAT2* or *P2RX5* between UCP1^histological+^ and UCP1^histological-^ samples, even though *PAT2* expression appeared higher in the UCP1^histological+^ samples (Fig. 2C,D). Furthermore, the expression of the classical brown specific markers *ZIC1* and *LHX8* were not different between UCP1^histological+^ and UCP1^histological-^ samples (Fig. 2E,F). To test for beige adipocyte specificity, we analyzed the expression of *TMEM26*, *HOXC9* and *TBX1*. While *TMEM26* and *HOXC9* did not show differences between UCP1^histological+^ and UCP1^histological-^ samples (Fig. 2G,H), *TBX1* mRNA was significantly decreased in UCP1^histological+^ samples compared to UCP1^histological-^ samples of lean children (Fig. 2I). Furthermore, UCP1^histological+^ samples showed significantly decreased *LEP* expression, a white adipocyte specific marker, compared to UCP1^histological-^ samples of children and adults (Fig. 2J), while *ASC1* (Fig. 2K), another WAT marker, was not different between UCP1^histological+^ and UCP1^histological-^ samples. We did not observe significant differences in the expression of the AT marker *ADIPOQ* (Fig. 2L) between UCP1^histological+^ and UCP1^histological-^ samples.

Comparison of expression levels of these molecular markers within the distinct UCP1^histological+^ samples did not reveal obvious differences in *UCP1*, *PRDM16*, *PAT2*, *P2RX5*, *ZIC1*, and *LHX8* expression between subcutaneous, perirenal and visceral depots (S1A–F Fig.), even though we did detect individual samples with exceedingly higher expression of *PAT2* or *P2RX5* in perirenal UCP1^histological+^ depots. Also, the expression of the beige adipocyte markers *TMEM26*, *HOXC9* or *TBX1*, and white adipocyte markers *LEP* or *ASC1* and AT marker *ADIPOQ*, were not different between subcutaneous, perirenal and visceral depots of UCP1^histological+^ depots (S2G–L Fig.). To expand our analyses to a more comprehensive picture of molecular markers, we employed data from Illumina BeadChip mircorarrays (S3 Table). *PGC1α* (*Peroxisome proliferator-activated receptor gamma coactivator 1-alpha*) and *CIDEA* (*Cell death activator CIDE-A*), markers for both brown and beige adipocytes, were significantly increased in UCP1^histological+^ samples (S2A,B Fig.). Of further beige markers, only *CAR4* (Carbonic Anhydrase IV) expression could be evaluated, and was significantly elevated in UCP1^histological+^ samples (S2C Fig.), whereas the signal of *MYF5*, *DIO2* and *CITED1* was below the detection limit and could not be evaluated. Hence, overall UCP1^histological+^ samples had a higher expression of *UCP1*, *PRDM16*, *PGC1α* and *CIDEA* compared to UCP1^histological-^ samples, but did not differ in the expression of beige specific markers such as *TMEM26* or *HOXC9*.

**Fig 3:**
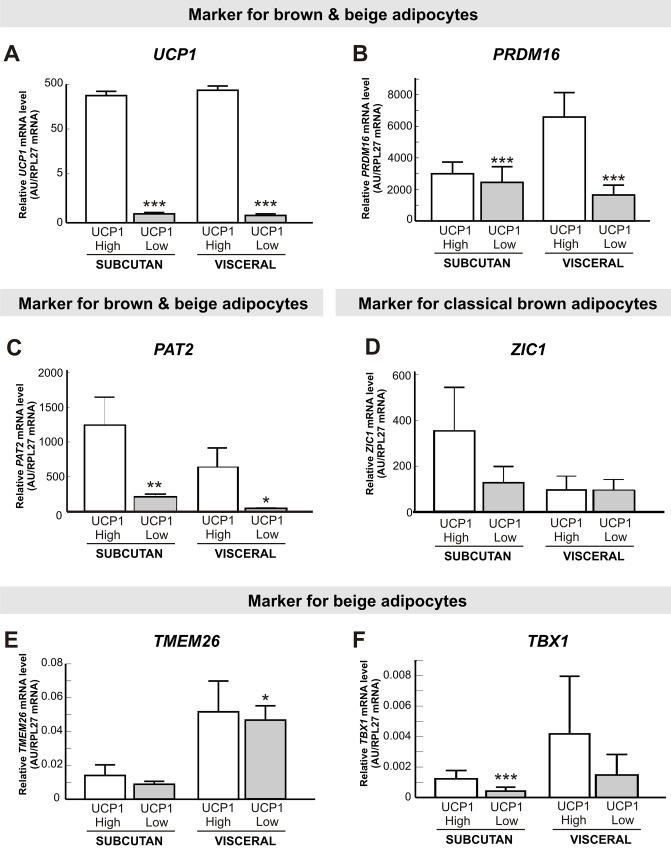
Expression analyses of brown and beige markers in adipose tissue samples of adults with low compared to high *UCP1* mRNA expression. We compared the upper 10% with the lowest 10% of *UCP1* mRNA expression in paired subcutaneous and visceral AT samples of adults (n = 48) (A). Subcutaneous and visceral AT samples of adults with a high *UCP1* expression showed significantly increased mRNA levels of *PRDM16* (B) and *PAT2* (C) in both subcutaneous and visceral depots. The classical brown specific marker *ZIC1* was not altered between subcutaneous/visceral samples with a low or high *UCP1* expression (D). Furthermore, samples with a high *UCP1* expression appear to have slightly higher *TMEM26* expression in subcutaneous and significantly increased mRNA levels in visceral AT samples (E). In contrast, *TBX1* levels were significant increased in subcutaneous depots and slightly in visceral depots (F). Expression levels were normalized with the reference gene *RPL27*. Statistical significance was assessed by Student t-test and was marked with *P<0.05; **P< 0.01; ***P<0.001.

To analyse a higher number of visceral AT samples and to expand our analyses to the adult age range, we provide data on *UCP1*, *PRDM16*, *PAT2*, *ZIC1*, *TMEM26* and *TBX1* mRNA expression from paired visceral and subcutaneous samples of lean and obese adults from a distinct adult cohort (Table 2). To analyse these data in a similar way as the childhood cohort, we compared the expression of brown and/or beige markers between the 10% with highest (UCP1 High) and the lowest 10% (UCP1 Low) of *UCP1* mRNA expression in subcutaneous and visceral biopsies (Fig. 3A). In line with the data in children, *PRDM16* expression was significantly increased in subcutaneous and visceral UCP1 High samples, but not at the same magnitude as *UCP1* (Fig. 3B). Furthermore, *PAT2* expression was significantly increased in UCP1 High samples in subcutaneous as well as in visceral biopsies (Fig. 3C). The expression of the classical brown adipocyte marker *ZIC1* was not altered between samples with high and low UCP1 expression (Fig. 3D). Expression of the beige marker *TMEM26* was not statistically significantly increased in subcutaneous samples of high *UCP1* expression, but was significantly higher expressed in UCP1 High visceral samples (Fig. 3E). *TBX1* mRNA appeared to be higher in UCP1 High samples in subcutaneous samples, but not in visceral samples (Fig. 3F). The results from the gene expression analyses are partly in line with our observation in the childhood cohort, with coherent results regarding the beige/brown and classical brown expression markers, while the more beige-specific marker *TBX1*, appear to be higher expressed in UCP1 High samples in adults, but not in children.

### Association of *UCP1* mRNA expression with anthropometric and molecular markers

**Fig 4:**
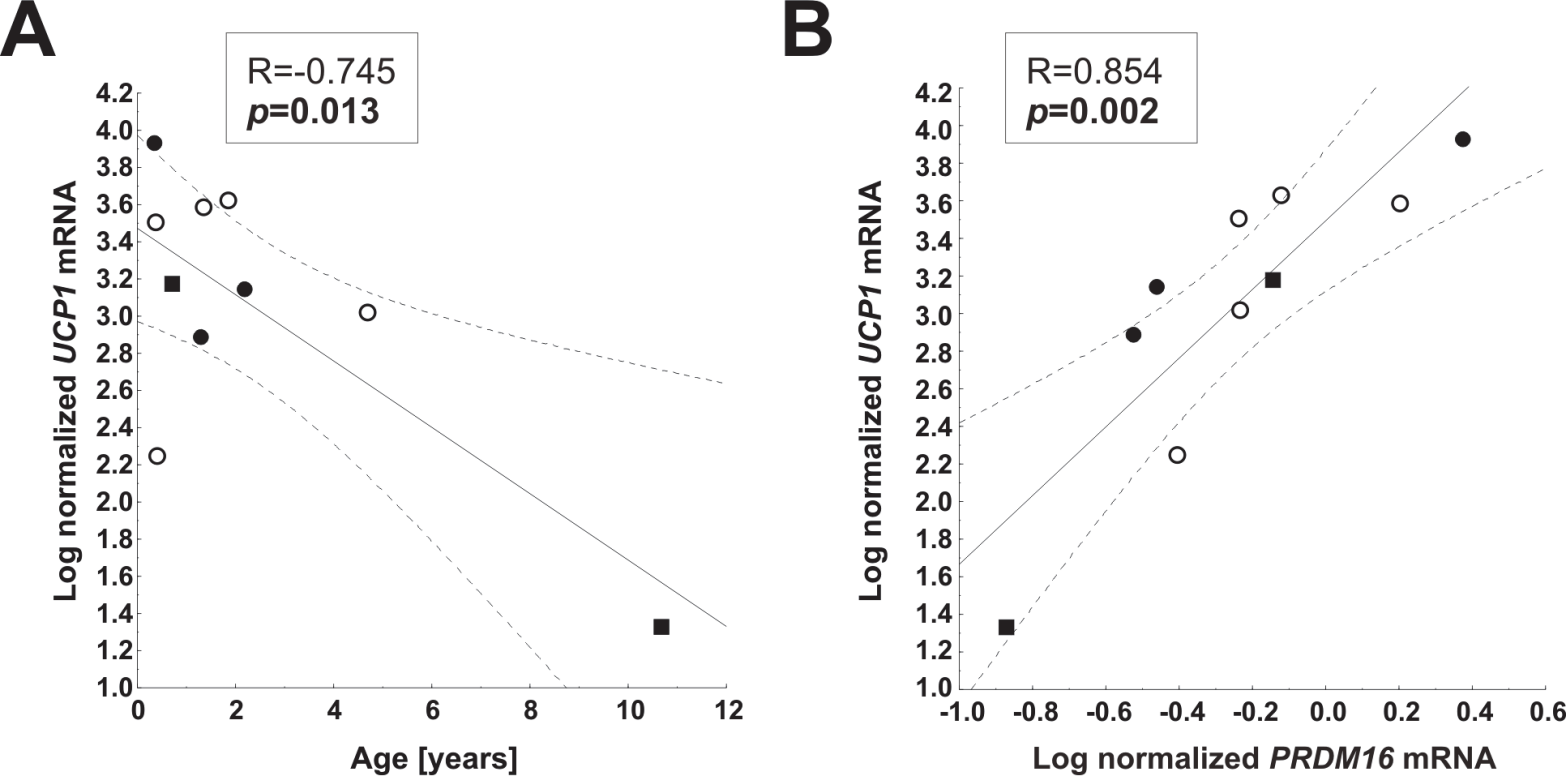
Correlation analysis in UCP1^histological+^ samples. In statistical analyses which were restricted UCP1^histological+^ samples, we observed a negative association between *UCP1* mRNA and age (A) and a positive association between *UCP1* mRNA and *PRDM16* (B). Pearson correlation coefficient R and *p*-value are shown in each scatterplot. Significant *p*-values (p<0.05) are indicated in bold. Black circles, subcutaneous depots; white circles, perirenal depots; and black squares, visceral depots.

We assessed potential associations of *UCP1* expression in AT with physical development in children. *UCP1* expression in subcutaneous UCP1^histological-^ did not correlate with anthropometric parameters of children (S4 Table). There were no correlations between *UCP1* mRNA levels with mean outside temperature, adipocyte size or expression of molecular markers. Since subjects in lean and obese groups were different with respect to mean age and BMI-SDS, we additionally controlled for age and BMI-SDS in partial correlation analyses, but again did not observe any associations (S4 Table). When we restricted our analyses to UCP1^histological+^ samples, there was a nominal weak negative correlation between UCP1 expression and age; however, as obvious from the graph, this correlation is attributed to a single subject (Fig. 4A). We detected a significant positive correlation of *UCP1* with *PRDM16* (Fig. 4B), but not with any other brown or beige adipocyte marker or with adipocyte size (Table 4) in UCP1^histological+^ samples. UCP1 expression was also not related to outside temperature (Table 4).

**Table 4:** Correlation of *UCP1* mRNA expression with anthropometric parameters and expression data in UCP1^histological+^ samples from children (n = 10).

Parameter	R	*p*
***Anthropometric parameters***		
Age [years]	**-0.75**	**0.013**
BMI-SDS	-0.07	>0.1
PH	-0.07	>0.1
Outside Temperature [°C]	-0.44	>0.1
***Biological data***		
Adipocyte area [μm²]	-0.47	>0.1
PRDM16 mRNA^a^	**0.85**	**0.002**
PAT2 mRNA^a^	0.51	>0.1
TMEM26 mRNA^a^	0.35	>0.1
TBX1 mRNA^a^	0.03	>0.1
HOXC9 mRNA^a^	0.61	0.083
P2RX5 mRNA^a^	0.40	>0.1
ZIC1 mRNA^a^	-0.2	>0.1
LHX8 mRNA^a^	-0.29	>0.1
LEP mRNA^a^	-0.12	>0.1
ASC1 mRNA^a^	0.36	>0.1
ADIPOQ mRNA^a^	0.46	>0.1

BMI, body-mass index; SDS, standard deviation score; PH, pubertal stage.

^a^Pearson correlation analysis was performed for log-transformed *UCP1* expression levels.

## Discussion

BAT is of importance in the adaptation to cold environment in humans, particularly in the newborn period [7]. Beyond infancy, BAT may also be of physiological relevance in the context of obesity through its capability to burn excess energy. This notion has been nourished by recent findings of BAT activity in imaging studies in adults [3,4,10]. Here we provide evidence for the presence of brown-like adipocytes in AT of children across a wide age span, which were free of major illness. Our results show that brown-like adipocytes i) were present in non-obese children well beyond first year of life, and ii) the presence was not confined to typical perirenal or visceral depots, but also interspersed within subcutaneous WAT depots.

According to classical concepts, BAT develops from the 5^th^ gestational week onwards, peaking around birth and declines over the next 9 months of extrauterine life [6,32]. In our study, the presence of brown-like adipocytes was not restricted to infants, as we identified them in samples of children up to 10 years of age. We cannot speculate on a potential age dependency in later life as our sample size of UCP1^histological+^ samples is too small and the weak negative correlation between *UCP1* mRNA and age seemed to be attributed to one subject.

Considering the effect on energy dissipation, variations in BAT activity may affect energy balance and even the development of obesity as has been shown for rodents [33,34]. In our study, brown-like adipocytes exclusively occurred in non-obese children, which is in agreement with observations of an inverse correlation between BAT presence and body weight from PET/CT studies [3,35]. However, our study may have been biased by the fact that obese children were slightly older and, more importantly, that we only had access to perirenal/visceral samples of lean children, which is likely to limit the interpretation on the presence of brown-like adipocytes in obese samples. Nevertheless, also in subcutaneous samples (where we had a larger number of samples from obese children), we did exclusively find brown-like adipocyte in lean children. Furthermore, one group showed that BAT presence rises with the progression of puberty [26], which would favor the obese group as they are more pubertally advanced.

In humans, most BAT is localized in the neck and along the spine as visualized by PET/CT studies in adults [3,4,17,36]. Furthermore, one study found BAT in the interscapular region of infants using imaging and histological methods [17]. In addition to these “brown fat depots”, WAT of mice and adult humans can contain cells with characteristics of brown adipocytes [37]. In our study, we detected brown-like adipocytes in WAT of perirenal, visceral and also interspersed within inguinal subcutaneous depots. At this point it is not entirely clear, where those brown-like adipocytes within WAT derive from. Possibilities include that brown-like adipocytes in WAT i) may originate from the maturation of designated brown adipocyte precursor cells [38], ii) differentiate from white preadipocytes, or iii) transdifferentiated from white adipocytes [2,38–40], so called “beige” cells [38,41,42]. To address the hypothesis that such beige adipocytes are responsible for the brown-like phenotype, we evaluated the expression of different markers specific for both brown and beige adipocytes (*UCP1*, *PRDM16*, *PAT2*, *P2RX5*, *PGC1α* and *CIDEA*), for classical brown adipocytes (*ZIC1* and *LHX8*) and beige adipocytes (*TMEM26*, *TBX1*, *HOXC9* and *CAR4*). In our cohort, those samples with histological and immunohistochemical evidence for brown-like adipocytes had a higher expression of *UCP1*, *PRDM16*, *PGC1α* and *CIDEA* compared to negative samples. They did, however, not differ in the expression of most beige selective markers, except for *CAR4*. We are thus, not able to provide evidence that beige adipocytes are responsible for the brown-like phenotype. When we expanded our analyses to another adult AT cohort, which we stratified for the samples with the highest and lowest *UCP1* expression in visceral and subcutaneous depots, the results of *PRDM16*, *PAT2*, *ZIC1* and *TMEM26* expression are in line with the data from our childhood cohort. Only *TBX1* was significantly increased in subcutaneous and slightly higher in visceral UCP1 High samples of adults. This is in contrast to our observation in childhood samples. However, also in the study by Lidell *et al*., *TBX1* was lower in the interscapular (and hence “classical brown”) depot compared to other depots [17]. It has to be mentioned that, while many of these marker genes have been useful for the characterization of specific isolated adipocyte subtypes, their usefulness in discriminating distinct adipocyte types in intact AT is uncertain. Furthermore, BAT is a highly dynamic tissue and it often contains a mixture of brown and white adipocytes. In humans, it is difficult to obtain "pure BAT depots" which are free of white adipocytes. Secondly, the specificity of some markers is still debated, and many markers are not exclusively expressed in one adipocyte subtype [43].

The observed prevalence of brown-like adipocytes in 7.6% in all children (or 10.3% in lean children) in our study may appear lower than expected from PET/CT imaging studies in children, where detection rates varied between <20% [15,44] to 44% [13] of activated BAT. There are several potential explanations for this. First: The observed discrepancy may be due to differences in the methodology and collected depots. PET/CT imaging studies usually screened the whole body for metabolically active BAT as opposed to the very tiny volumes of AT samples we had available from biopsies in our study. Secondly, these studies indicate that most of the BAT is localized in the cervical-supraclavicular area of the body [3,4,36]. AT samples from our cohort were mainly taken from the lower body half. Third, the study population in our study consists of children who are free of major disease. This may be of relevance, as it was shown that cancer-associated cachexia patients exhibited WAT browning in the initial stages of cancer-associated cachexia, suggesting a higher BAT prevalence in cancer patients [45]. Finally, even though 100% of visceral depots and 83% of perirenal depots showed brown-like adipocytes, our cohort consists predominantly of subcutaneous biopsies, in which minute islets of brown-like adipocytes are scattered within classical WAT. Also, the small sample volume of AT, particularly in subcutaneous biopsies, may contribute to the lack of detection of brown-like adipocytes. However, the concordance between UCP1 detection in immunohistochemistry and mRNA expression is reassuring. Only one UCP1^histological-^ sample revealed a high *UCP1* expression reaching the level observed in the UCP1^histological+^ samples. A further consideration is that most PET/CT examinations were based on retrospective, cross-sectional studies of subjects treated for malignancy, which was an exclusion criterium in our study [21].

BAT is known to be activated by cold and some adult studies documented a negative correlation between BAT and outside temperature [3]. Also, outdoor workers in Finland were shown to have increased BAT depots [46]. We were not able to document an association with outside temperature or season in children, which is in line with another childhood imaging study [13].

One limitation of our study is that our AT samples consist primarily of subcutaneous depots, which might contribute to the low detection rate of BAT. Furthermore, UCP1^histological-^ samples are derived from different depots from children and we did not have access to cervical and supraclavicular samples, where the highest BAT detection rates have been described in childhood imaging studies compared to abdominal and paraspinal locations [13]. The small number of AT samples containing brown-like adipocytes and the cross-sectional study design may contribute to the lack of potential associations with age and obesity [13]. On the other hand, we describe a more direct evidence of brown or brown-like adipocytes on the histological and molecular level. Also, the children of our cohort were free of metabolic or malignant illness and did not take medication, whereas the imaging studies were mainly performed in pediatric oncology patients or deceased children.

In summary, we provide evidence for the presence of brown-like adipocytes in WAT depots in children at the level of the AT applying histological and molecular approaches. Our data indicate that brown-like adipocytes are present well beyond the first year of life in non-obese children. The presences of brown-like adipocytes was not confined to typical locations but were also detectable in visceral and interspersed in subcutaneous WAT.

## Supporting Information

S1 Fig Expression analyses of brown, beige, and white adipocyte markers in UCP1^histological+^ samples of different depots. The comparison of expression levels of brown adipocyte markers within UCP1^histological+^ depots did not reveal obvious differences in the expression of *UCP1* (A), *PRDM16* (B), *PAT2* (C), *P2RX5* (D), *ZIC1* (E), and *LHX8* (F) between subcutaneous, perirenal and visceral depots. Furthermore, the expression levels of beige adipocyte markers *TMEM26* (G), *TBX1* (H) and *HOXC9* (I) and the expression levels of white adipocyte markers like *LEP* (J) and *ASC1* (K) were not different between subcutaneous, perirenal and visceral depots of UCP1^histological+^ depots. In addition, *ADIPOQ* (L) expression was not different between subcutaneous, perirenal and visceral depots of UCP1^histological+^ depots. However, *UCP1* expression was clearly increased in all depots of UCP1^histological+^ samples compared to UCP1^histological-^ subcutaneous samples. All UCP1^histological+^ samples, regardless of the depot, showed decreased *LEP* mRNA levels compared to UCP1^histological-^ subcutaneous samples. Target gene expression was normalized to the mean of the three housekeeping genes: *ACTB*, *TBP* and *HPRT1*. Data are presented as mean ± SEM.
(TIF)

S2 Fig Expression analysis of brown and beige adipocyte markers from Illumina BeadChip mircorarrays. Adipose tissue mRNA levels of *PGC1 α* (A) and *CIDEA* (B), markers for both beige and brown adipocytes were significantly increased in UCP1^histological+^ samples compared to UCP1^histological-^ samples of lean or obese children. Furthermore, also the beige marker *CAR4* (C) was significantly increased in UCP1 positive samples compared to UCP1 negative samples of lean or obese children. Data are presented as mean ± SEM. *, *p*<0.05; **, *p*<0.01; ***, p<0.001. Statistical significance was assessed by a one-way ANOVA with a post-hoc Dunnett’s test.
(TIF)

S1 Table Human qRT-PCR primer sequences. UCP1, Uncoupling protein-1; PRDM16, PR domain containing 16; PAT2, Proton assistant amino acid transporter-2; P2RX5, Purinergic receptor P2X, ligand-gated ion channel 5; ZIC1, Zinc finger protein of the cerebellum 1; TMEM26, Transmembrane Protein 26; HOXC9, Homeobox C9; ASC1, Asc-type amino acid transporter 1; ADIPOQ, Adiponectin; HPRT1, Hypoxanthine phosphoribosyltransferase 1; ACTB, ß-Actin; TBP, TATA box binding protein; RPL27, Ribosomal protein L27.
(DOCX)

S2 Table Taq Man Assays used in qRT-PCR for characterization of human AT. LHX8, LIM homeobox 8; TBX1, T-box 1; LEP, Leptin.; PRDM16, PR domain containing 16; TMEM26, Transmembrane Protein 26.
(DOCX)

S3 Table Characteristics of children from Illumina BeadChip mircorarray analysis. Data are given as mean ± SEM (range). For gender, statistical significance was analysed by chi square test. Statistical significance for differences between lean and obese individuals was determined by Students t-test. Significant *p*-values are indicated in bold. BMI, body-mass index; SDS, standard deviation score; PH, pubertal stage.
(DOCX)

S4 Table Correlation of *UCP1* mRNA expression with anthropometric parameters and expression of molecular markers in subcutaneous UCP1^histological-^samples from children [n = 121]. BMI, body-mass index; SDS, standard deviation score; PH, pubertal stage. ^a^Pearson correlation analysis was performed for log- transformed UCP1 expression levels. ^b^Partial correlation analysis after adjustment for age and BMI SDS.
(DOC)
