# Supplementary figures and images for: Direct Evidence of Brown Adipocytes in Different Fat Depots in Children
Source: PLoS One. 2015 Feb 23;10(2):e0117841. doi: 10.1371/journal.pone.0117841 (PMC4338084; doi:10.1371/journal.pone.0117841)

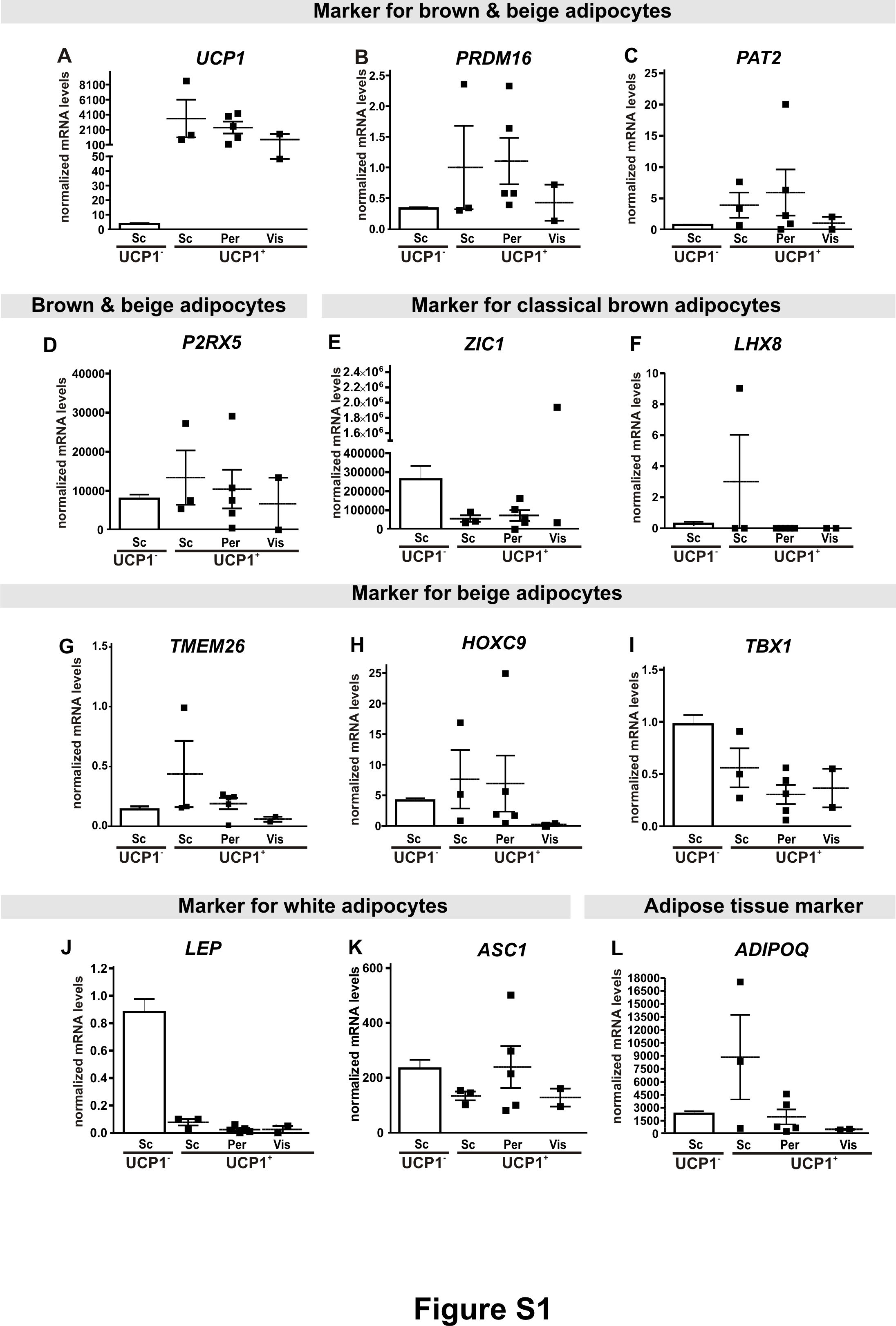

Supplement: S1 Fig — The comparison of expression levels of brown adipocyte markers within UCP1histological+ depots did not reveal obvious differences in the expression of UCP1 (A), PRDM16 (B), PAT2 (C), P2RX5 (D), ZIC1 (E), and LHX8 (F) between subcutaneous, perirenal and visceral depots. Furthermore, the expression levels of beige adipocyte markers TMEM26 (G), TBX1 (H) and HOXC9 (I) and the expression levels of white adipocyte markers like LEP (J) and ASC1 (K) were not different between subcutaneous, perirenal and visceral depots of UCP1histological+ depots. In addition, ADIPOQ (L) expression was not different between subcutaneous, perirenal and visceral depots of UCP1histological+ depots. However, UCP1 expression was clearly increased in all depots of UCP1histological+ samples compared to UCP1histological- subcutaneous samples. All UCP1histological+ samples, regardless of the depot, showed decreased LEP mRNA levels compared to UCP1histological- subcutaneous samples. Target gene expression was normalized to the mean of the three housekeeping genes: ACTB, TBP and HPRT1. Data are presented as mean ± SEM. (TIF) [file pone.0117841.s001.tif]

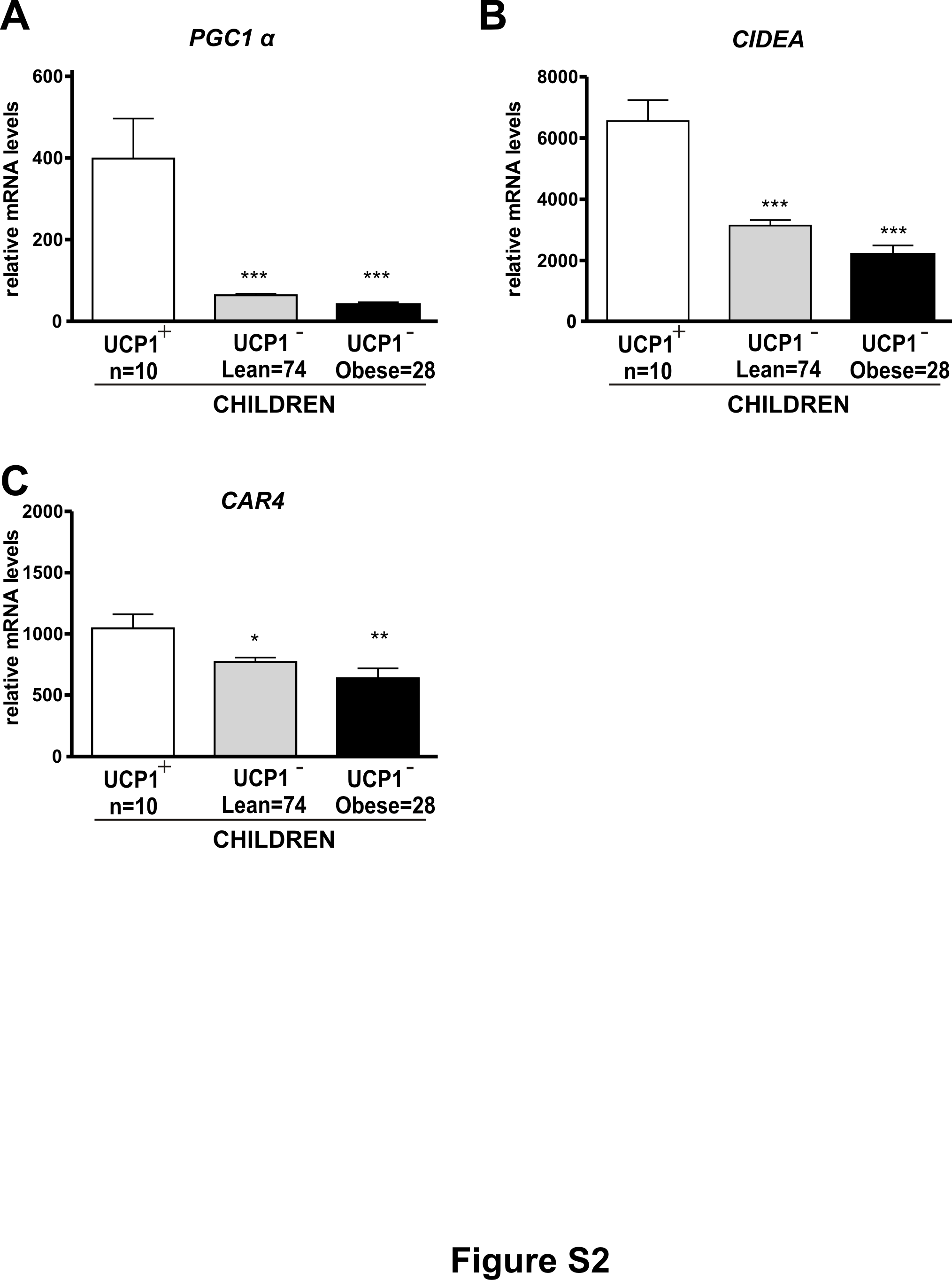

Supplement: S2 Fig — Adipose tissue mRNA levels of PGC1 α (A) and CIDEA (B), markers for both beige and brown adipocytes were significantly increased in UCP1histological+ samples compared to UCP1histological- samples of lean or obese children. Furthermore, also the beige marker CAR4 (C) was significantly increased in UCP1 positive samples compared to UCP1 negative samples of lean or obese children. Data are presented as mean ± SEM. *, p<0.05; **, p<0.01; ***, p<0.001. Statistical significance was assessed by a one-way ANOVA with a post-hoc Dunnett’s test. (TIF) [file pone.0117841.s002.tif]
