# Supplementary material for: Direct Evidence of Brown Adipocytes in Different Fat Depots in Children
Source: PLoS One. 2015 Feb 23;10(2):e0117841. doi: 10.1371/journal.pone.0117841 (PMC4338084; doi:10.1371/journal.pone.0117841)
Supplement: S1 Table — UCP1, Uncoupling protein-1; PRDM16, PR domain containing 16; PAT2, Proton assistant amino acid transporter-2; P2RX5, Purinergic receptor P2X, ligand-gated ion channel 5; ZIC1, Zinc finger protein of the cerebellum 1; TMEM26, Transmembrane Protein 26; HOXC9, Homeobox C9; ASC1, Asc-type amino acid transporter 1; ADIPOQ, Adiponectin; HPRT1, Hypoxanthine phosphoribosyltransferase 1; ACTB, ß-Actin; TBP, TATA box binding protein; RPL27, Ribosomal protein L27. (DOCX) [file pone.0117841.s003.docx]

| **Table S1. Human qRT-PCR primer sequences.** | | |
| --- | --- | --- |
| **Gene** | **Forward Primer** | **Probe 5`-3`** |
| **UCP1** | F 5´- ACGACACGGTCCAGGAGTTC-3`  R 5´- ACCAGCTAAAATCTTGCTTCCTAAAC-3` | TCACCGCAGGGAAAGAAACAGCACC |
| **PRDM16** | F 5´- CCAATAGTGAGATGAACCAAGCAT-3`  R 5´- CCGTCCACGATCTGCATGT-3` | AACGCGAACAGAGAAACGGGCG |
| **PAT2** | F 5- CCTGCCACTGTATGCACATC-3`  R 5´- TAGTCCATGCATCACCGTGT-3` |  |
| **P2RX5** | F 5´- TTCACCAACACCTCGGATCT-3`  R 5´- CAGGTTGGTGACCACAAAAA-3 |  |
| **ZIC1** | F 5´- AAGATCCACAAAAGGACGCA-3` R 5´- CACGTGCATGTGCTTCTTG-3` |  |
| **TMEM26** | F 5´- AGCATGCTGCAGTTTCCACTT-3`  R 5´- TCACAGACACAGGGCACACAA-3` | CCTGGCAGTACAGAAC |
| **HOXC9** | F 5´- CGGCAGCAAGCACAAAGAG-3`  R 5´- CGGTCCCTGGTTAAATACATATTGA-3` | CCGACCTGGACCCCAGCAACC |
| **ASC1** | F 5´- GTGGCGCTCAAGAAGGAGAT-3`  R 5´- CCTTGGGCGAGATGAAGAT-3` |  |
| **ADIPOQ** | F 5´- GGCCGTGATGGCAGAGAT-3`  R 5´- CCTTCAGCCCCGGGTACT-3` | GATGTCTCCCTTAGGACCAATAAGACCT GG |
| **HPRT1** | F 5´- GGCAGTATAATCCAAAGATGGTCAA-3`  R 5´- GTCTGGCTTATATCCAACACTTCGT-3` | CAAGCTTGCTGGTGAAAAGGACCCC |
| **ACTB** | F 5´- CGACGCGGCTACAGCTT-3`  R 5´- CCTTAATGTCACGCACGATTT-3` | ACCACCACGGCCGAGCGG |
| **TBP** | F 5´- TTGTAAACTTGACCTAAAGACCATTGC-3`  R 5´- TTCGTGGCTCTCTTATCCTCATG-3` | AACGCCGAATATAATCCCAAGCGGTTG´ |
| **qRT-PCR primer sequences for the further adult cohort** | | |
| **UCP1** | F 5´- GTCTCGAATCTTTGAATCAAATC-3`  R 5´- CTGTGTTAACAGTTACAGCC-3` | TCCATAACCGCAGCCATCCATCCA |
| **RPL27** | F 5´- GCTGTCATCGTGAAGAAC-3`  R 5´- CTTGGCGATCTTCTTCTT-3` | CGGTCAATTCCAGCCACCAGAGC |

UCP1, Uncoupling protein-1; PRDM16, PR domain containing 16; PAT2, Proton assistant amino acid transporter-2; P2RX5, Purinergic receptor P2X, ligand-gated ion channel 5; ZIC1, Zinc finger protein of the cerebellum 1; TMEM26, Transmembrane Protein 26; HOXC9, Homeobox C9; ASC1, Asc-type amino acid transporter 1; ADIPOQ, Adiponectin; HPRT1, Hypoxanthine phosphoribosyltransferase 1; ACTB, ß-Actin; TBP, TATA box binding protein; RPL27, Ribosomal protein L27.
