# Supplementary material for: Direct Evidence of Brown Adipocytes in Different Fat Depots in Children
Source: PLoS One. 2015 Feb 23;10(2):e0117841. doi: 10.1371/journal.pone.0117841 (PMC4338084; doi:10.1371/journal.pone.0117841)
Supplement: S2 Table — LHX8, LIM homeobox 8; TBX1, T-box 1; LEP, Leptin.; PRDM16, PR domain containing 16; TMEM26, Transmembrane Protein 26. (DOCX) [file pone.0117841.s004.docx]

| **Table S2. Taq Man Assays used in qRT-PCR for charcterisation of human AT.** | | |
| --- | --- | --- |
| **Gene** | **Assay ID** | **Reference Sequence** |
| **LHX8** | Hs00418293_m1 | NM_001001933.1 |
| **TBX1** | Hs00962556_m1 | NM_005992.1 |
| **LEP** | Hs00174877_m1 | NM_000230.2 |
| **Taq Man Assays for the further adult cohort** | | |
| **PRDM16** | HS 00223161 m1 | NM_022114.3 |
| **TMEM26** | Hs00415619_m1 | NM_178505.6 |

LHX8, LIM homeobox 8; TBX1, T-box 1; LEP, Leptin.; PRDM16, PR domain containing 16; TMEM26, Transmembrane Protein 26.
