# Supplementary material for: Direct Evidence of Brown Adipocytes in Different Fat Depots in Children
Source: PLoS One. 2015 Feb 23;10(2):e0117841. doi: 10.1371/journal.pone.0117841 (PMC4338084; doi:10.1371/journal.pone.0117841)
Supplement: S3 Table — Data are given as mean ± SEM (range). For gender, statistical significance was analysed by chi square test. Statistical significance for differences between lean and obese individuals was determined by Students t-test. Significant p-values are indicated in bold. BMI, body-mass index; SDS, standard deviation score; PH, pubertal stage. (DOCX) [file pone.0117841.s005.docx]

| **Table S3. Characteristics of children from Illumina BeadChip mircorarray analysis.** | | | |
| --- | --- | --- | --- |
|  | **Lean** | **Obese** | ***p*** |
| n | 84 | 28 |  |
| female/male [n] | 33/51 | 11/17 | 0.823 |
| Age [years] | 8.3 ± 0.6  (0.2 – 20.7) | 7.4 ± 0.8  (0.3 – 12.8) | 0.495 |
| BMI-SDS | -0.42 ± 0.1  (-4.6 – 1.2) | 2.3 ± 0.1  (1.3 – 3.6) | **<0.001** |
| PH | 2  (1 – 6) | 1  (1 – 6) | **0.043** |
| **Depots** |  |  |  |
| Subcutaneous | 75 | 27 |  |
| Perirenal | 7 | 1 |  |
| Visceral | 2 | 0 |  |

Data are given as mean ± SEM (range). For gender, statistical significance was analysed by chi square test. Statistical significance for differences between lean and obese individuals was determined by Students t-test. Significant *p*-values are indicated in bold. BMI, body-mass index; SDS, standard deviation score; PH, pubertal stage.
