# Supplementary material for: Direct Evidence of Brown Adipocytes in Different Fat Depots in Children
Source: PLoS One. 2015 Feb 23;10(2):e0117841. doi: 10.1371/journal.pone.0117841 (PMC4338084; doi:10.1371/journal.pone.0117841)
Supplement: S4 Table — BMI, body-mass index; SDS, standard deviation score; PH, pubertal stage. aPearson correlation analysis was performed for log- transformed UCP1 expression levels. bPartial correlation analysis after adjustment for age and BMI SDS. (DOC) [file pone.0117841.s006.doc]

| **Table S4. Correlation of *UCP1* mRNA expression with anthropometric parameters and expression of molecular markers in subcutaneous UCP1^histological -^ samples from children [n=121].** | | | | | | |
| --- | --- | --- | --- | --- | --- | --- |
| **Parameter** | **R** | | | ***p*** | **R^b^** | ***p^b^*** |
| ***Anthropometric parameters*** | | | | | | |
| Age [years] | | -0.13 | | > 0.1 |  |  |
| BMI-SDS | | -0.05 | | > 0.1 |  |  |
| PH | | - 0.06 | | > 0.1 | 0.05 | > 0.1 |
| Outside Temperature [°C] | | | 0.23 | > 0.1 | 0.05 | > 0.1 |
|  | |  | |  |  |  |
| ***Biological data*** | |  | |  |  |  |
| Adipocyte area [µm²] | | | -0.054 | > 0.1 | -0.07 | > 0.1 |
| *PRDM16* mRNA^a^ | | | 0.04 | > 0.1 | -0.04 | > 0.1 |
| *PAT2* mRNA^a^ | | | -0.05 | > 0.1 | -0.06 | > 0.1 |
| *P2RX5* mRNA^a^ | | | -0.09 | > 0.1 | -0.12 | > 0.1 |
| *ZIC1* mRNA^a^ | | | 0.11 | > 0.1 | 0.21 | > 0.1 |
| *LHX8* mRNA^a^ | | | -0.09 | > 0.1 | -0.19 | > 0.1 |
| *TMEM26* mRNA^a^ | | | 0.09 | > 0.1 | 0.08 | > 0.1 |
| *TBX1* mRNA^a^ | | | -0.04 | > 0.1 | -0.05 | > 0.1 |
| *HOXC9* mRNA^a^ | | | -0.01 | > 0.1 | -0.02 | > 0.1 |
| *LEP* mRNA^a^ | | | 0.12 | > 0.1 | 0.18 | > 0.1 |
| *ASC1* mRNA^a^ | | | 0.16 | > 0.1 | 0.14 | > 0.1 |
| *ADIPOQ* mRNA^a^ | | | 0.14 | > 0.1 | 0.13 | > 0.1 |

BMI, body-mass index; SDS, standard deviation score; PH, pubertal stage. ^a^Pearson correlation analysis was performed for log‑ transformed UCP1 expression levels. ^b^Partial correlation analysis after adjustment for age and BMI SDS.
